# A Simple Method for the Measurement of Young’s Moduli of Bilayer Thin Films Based on the Electrostatic Drive Approach

**DOI:** 10.3390/mi13111943

**Published:** 2022-11-10

**Authors:** Haiyun Liu, Zhen Zhang, Hongmin Gao, Lili Zhang, Lei Wang

**Affiliations:** 1 College of Computer and Information, Hohai University, Nanjing 211100, China; 2 Key Laboratory of MEMS of the Ministry of Education, Southeast University, Nanjing 210096, China

**Keywords:** bilayer thin films, Young’s modulus, process control monitoring (PCM), electrostatic drive method

## Abstract

This paper presents a simple method for the in situ determination of Young’s moduli of surface-micromachined bilayer thin films. The test structure consists of a cantilever, a bottom drive electrode located near the anchor, and a bottom contact electrode placed below the free end of the cantilever. The cantilever is driven by applying a voltage sweep between the cantilever and the drive electrode, and bends due to the electrostatic force. A novel theoretical model is derived to relate Young’s modulus with the applied voltage and structure dimensions. The theoretical model is validated by finite element simulation. Test structures for Au/polysilicon thin films are fabricated by the PolyMUMPsand tested with the current–voltage measurement system. The measured Young modulus of polysilicon ranges from 152.344 GPa to 154.752 GPa, and the measured Young modulus of Au ranges from 71.794 GPa to 74.880 GPa. Compared with existing extraction methods, the proposed method is featured with simple operation, good repeatability, relatively high precision, and low requirements for equipment. It can be used alongside the application of a process control monitor (PCM) in surface-micromachining process lines.

## 1. Introduction

Multilayered structures have been widely used in various types of MEMS devices, including RF MEMS devices [1], uncooled infrared cameras [2], micromirrors [3], speakers, pressure gauges, microphones, and other devices. The performance of MEMS is significantly affected by various material parameters, among which Young’s modulus is a critical material parameter in most MEMS devices [4,5,6,7,8]. The process control monitor (PCM) of Young’s moduli in the fabrication process of MEMS is an important part of ensuring the consistency and yield of MEMS products [9,10]. It is hoped that test methods can be performed at the wafer level using ordinary wafer–probe test equipment, since parameters extracted in such methods could be directly used in quality control. Most conventional methods for measuring Young’s modulus, such as nanoindentation, bulge test, tensile testing, etc., require the test wafer to be moved into the test instruments, which is inconvenient for high-volume manufacturing. The existing PCM extraction methods for Young’s modulus of thin films in MEMS mainly include the pull-in voltage method [6,11], the resonance frequency method [12,13,14,15,16,17,18], and the electrostatic drive method [19,20]. Among them, the pull-in voltage method is convenient for application but has poor repeatability; the resonant frequency method is featured with high resolution [21] and good repeatability, but the ring-down scheme is hard to deploy in the atmosphere and the phase-locking scheme will increase the complexity of the test equipment. Therefore, it is necessary to design a structure for the in situ measuring of Young’s moduli of multilayer films with good repeatability and low equipment requirements.

In this paper, a test structure based on electrostatic drive method is proposed for the determination of Young’s moduli of MEMS multilayer films. A theoretical model of the test structure is established and verified by simulation. Finally, bilayer test structures are fabricated as a typical example and experiments are carried out in the atmosphere. The experimental results agree well with the relevant literature data.

## 2. Theory

### 2.1. Structure Design

**Figure 1:**
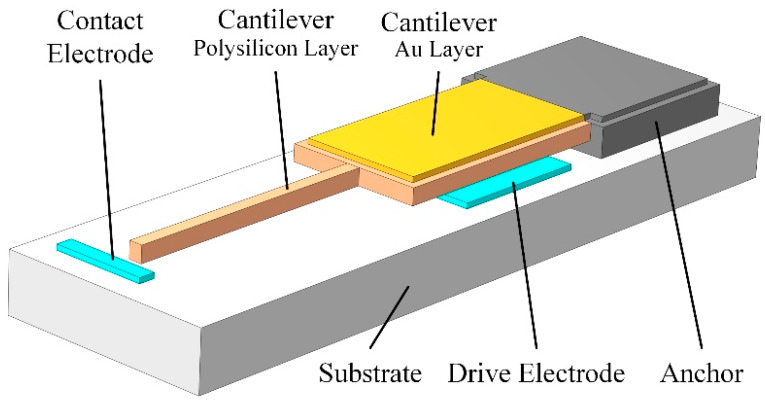
Schematic diagram of a test structure.

The test structure consists of a cantilever beam and two polysilicon bottom electrodes, wherein the cantilever beam is the film to be tested. The test structure for the bilayer film is composed of two similar structures. The cantilever beam of test structure A is a single-layer film, and the cantilever beam of test structure B is a bilayer film, as shown in Figure 1. The layout of test structure B is basically the same as that of test structure A, except that a new layer is deposited on the upper surface of the cantilever beam. Similar to the bilayer film, the test structure can be extended to three or more layers by using three or more structures.

**Figure 2:**
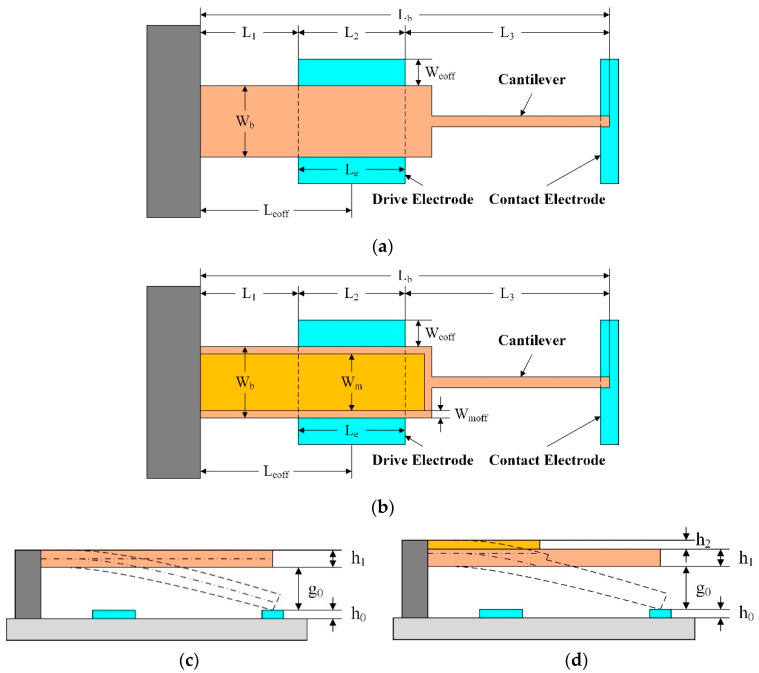
Dimensions in the test structure: (**a**) top view of test structure A; (**b**) top view of test structure B; (**c**) cross-sectional view of test structure A; and (**d**) cross-sectional view of test structure B.

Test structure A is shown in Figure 2a,c, and one end of the polysilicon cantilever beam of test structure A is fixed on the substrate via the anchor; a bottom drive electrode is fabricated on the substrate near the anchor; and a bottom contact electrode is fabricated on the substrate below the free end of the cantilever beam. The drive electrode is used to apply a driving voltage to produce electrostatic force, while the contact electrode is used to detect the contact at the free end of the cantilever beam. The length of the cantilever beam is *L_b_*, the width is *W_b_*, and the thickness is *h*_1_. The length of the drive electrode is *L_e_*; the width of the drive electrode is widened by *W_eoff_* at each edge than the width of the cantilever beam; the distance between the middle point of the drive electrode and the anchor is *L_eoff_*; and the thickness of the drive electrode is h_0_. The initial gap between the cantilever beam and the drive electrode is g_0_. In order to avoid the pull-in phenomenon, *L_eoff_* should be less than one third of L_b_. As shown in Figure 2b,d, the cantilever structure of test structure B is an Au/polysilicon bilayer film. The thickness of the Au layer is *h*_2_. Due to process constraints, both the length and width of the Au layer are smaller than those of the polysilicon layer. The distance between the edge of the Au layer and the polysilicon layer is *W_moff_*; accordingly, the width of the Au layer is *W_m_*. A different design dimension of test structure B with test structure A will not affect the data processing and calculations of Young’s moduli.

The cantilever beam, the drive electrode, and the contact electrode are made of materials with high electrical conductivity, and an insulator layer is deposited on the upper surface of the substrate to isolate the drive electrode and the contact electrode. The test structure has three terminals, which are connected to the cantilever beam, drive electrode, and contact electrode, respectively. When a voltage is applied between the cantilever beam and the drive electrode, the cantilever beam will bend due to the electrostatic force. As the driving voltage increases, the bending angle of the cantilever beam increases until the free end of the cantilever beam contacts the contact electrode. The effective Young modulus of the thin film material can be extracted from the driving voltage and structure dimensions by using the theoretical model.

### 2.2. Theoretical Model of Test Structure A with a Single-Layer Beam

For a single-layer cantilever beam, the general method for determining the beam deflection is to solve the quadratic differential equation of the beam:

(1) $$\frac{EI\left(d^{2}v\left(x\right)\right)}{dx^{2}}=M\left(x\right)$$

where *E* is Young’s modulus of the material, *I* is the moment of inertia of the cantilever beam, *M*(*x*) denotes the bending moment of the cross-section at point *x*, and *v*(*x*) represents the vertical displacement at point *x*.

As demonstrated in Figure 2a,b, *L*_1_, *L*_2_, and *L*_3_ are the lengths of three parts of the cantilever beam from left to right.

(2) $$\begin{array}{c} L_{1}=L_{eoff}-\frac{L_{e}}{2}\\ L_{2}=L_{e}\\ L_{3}=L_{b}-L_{eoff}-\frac{L_{e}}{2} \end{array}$$

The left side of the *L*_1_ region is connected to the anchor. The *L*_2_ region is subjected to the electrostatic force, and *L*_2_ is the same as *L_e_*. The *L*_3_ region is the part that will not be affected by the electrostatic force or the bending moment.

Since the beam deflection of the test structure is small, the deflection difference in the *L*_2_ region is ignored in our model. The electrostatic force between the bottom drive electrode and the cantilever beam is assumed as a uniformly distributed intensity *q*. The area of q on the cantilever beam is the projection of the bottom drive electrode. The bending angle at the free end of the cantilever beam (*θ_b_*) can be expressed as a function of *q* [22]:

(3) $$\theta_{b}=\theta_{2}=\frac{q}{6EI}\left[{\left(L_{1}+L_{2}\right)}^{3}-L_{1}^{3}\right]$$

*θ*_2_ represents the bending angle at the free end of *L*_2_ region, which is the same as *θ_b_*.

The vertical displacement at the free end of the cantilever beam (*δ_b_*) is given by:

(4) $$\begin{array}{ll} \delta_{b} & =\delta_{2}+\theta_{b}L_{3}\\ & =\frac{q}{24EI}\left[3{\left(L_{1}+L_{2}\right)}^{4}-4L_{1}^{3}\left(L_{1}+L_{2}\right)+L_{1}^{4}\right]+\frac{qL_{3}}{6EI}\left[{\left(L_{1}+L_{2}\right)}^{3}-L_{1}^{3}\right]\\ & =\frac{qL_{2}\left[8L_{1}^{3}+12L_{1}L_{2}\left(L_{2}+L_{3}\right)+6L_{1}^{2}\left(3L_{2}+2L_{3}\right)+L_{2}^{2}\left(3L_{2}+4L_{3}\right)\right]}{24EI} \end{array}$$

where *δ*_2_ is the vertical displacement at the free end of *L*_2_ region. *δ*_2_ is calculated using the classical formula from mechanics of materials [22].

When the free end of the cantilever beam is in contact with the contact electrode, *δ_b_* is equal to *g*_0_. Equation (4) can be rewritten as:

(5) $$g_{0}=\frac{qL_{2}\left[8L_{1}^{3}+12L_{1}L_{2}\left(L_{2}+L_{3}\right)+6L_{1}^{2}\left(3L_{2}+2L_{3}\right)+L_{2}^{2}\left(3L_{2}+4L_{3}\right)\right]}{24EI}$$

The moment of inertia of a single-layer cantilever beam is:

(6) $$I=\frac{1}{12}W_{b}h_{1}^{3}$$

The charges on the cantilever beam and bottom drive electrode are considered in the calculation of electrostatic force, while the charges on other parts are ignored. Therefore, the expression of intensity *q* induced by electrostatic force may be written as follows:

(7) $$q=W_{b}\frac{\varepsilon_{0}U^{2}}{2g^{2}}$$

where *g* is the gap between the cantilever beam and the drive electrode when the cantilever beam is bent, *ε*_0_ stands for the vacuum permittivity, and *U* denotes the driving voltage.

Since *q* is set to be a constant value, *q* can be obtained using Equation (5):

(8) $$q=\frac{24EIg_{0}}{L_{2}\left[8L_{1}^{3}+12L_{1}L_{2}\left(L_{2}+L_{3}\right)+6L_{1}^{2}\left(3L_{2}+2L_{3}\right)+L_{2}^{2}\left(3L_{2}+4L_{3}\right)\right]}$$

The vertical displacement [22] of the cantilever beam in the *L*_2_ region is described as:

(9) $$v\left(x\right)=-\frac{q}{24EI}\left[x^{4}-4\left(L_{1}+L_{2}\right)x^{3}+6{\left(L_{1}+L_{2}\right)}^{2}x^{2}-4L_{1}^{3}x+L_{1}^{4}\right]$$

In order to simplify the model, *g* is considered as a constant value due to a constant *q*. As a consequence, *v*(*x*) is also considered as a constant value *v_g_*. Note that the beam displacement is nonlinear and the moment of inertia varies at different locations of the *L*_2_ region, and the value of *v_g_* is approximate to the integral mean value of the cube of *v*(*x*) multiplied by *x* in the *L*_2_ region. By substituting Equation (8) into Equation (9), *v_g_* is estimated by:

(10) $$\begin{array}{ll} v_{g} & =\frac{{\left(\int_{L_{1}}^{L_{1}+L_{2}}v^{3}\left(x\right)\;xdx\right)}^{\frac{1}{3}}}{L_{2}\left(L_{1}+\frac{L_{2}}{2}\right)}\\ & =\frac{g_{0}{\left(a_{1}L_{1}^{10}+a_{2}L_{1}^{9}L_{2}+a_{3}L_{1}^{8}L_{2}^{2}+a_{4}L_{1}^{7}L_{2}^{3}+a_{5}L_{1}^{6}L_{2}^{4}+a_{6}L_{1}^{5}L_{2}^{5}+a_{7}L_{1}^{4}L_{2}^{6}+a_{8}L_{1}^{3}L_{2}^{7}+a_{9}L_{1}^{2}L_{2}^{8}+a_{10}L_{1}L_{2}^{9}+a_{11}L_{2}^{10}\right)}^{\frac{1}{3}}}{{\left(L_{1}+\frac{L_{2}}{2}\right)}^{\frac{1}{3}}\left[8L_{1}^{3}+12L_{1}L_{2}\left(L_{2}+L_{3}\right)+6L_{1}^{2}\left(3L_{2}+2L_{3}\right)+L_{2}^{2}\left(3L_{2}+4L_{3}\right)\right]} \end{array}$$

The values of the constants in Equation (10) are shown in Table 1.

The gap g between the drive electrode and the cantilever is:

(11) $$g=g_{0}-v_{g}$$

The driving voltage *U* is gradually increased and the current between the cantilever beam and the contact electrode is monitored. When the current indicates conduction, the driving voltage is recorded as *U_CA_*. Young’s modulus *E* can be obtained by substituting Equation (8) and Equation (11) into Equation (7):

(12) $$E=W_{b}\frac{L_{2}\left(8L_{1}^{3}+18L_{1}^{2}L_{2}+12L_{1}L_{2}^{2}+3L_{2}^{3}+12L_{1}^{2}L_{3}+12L_{1}L_{2}L_{3}+4L_{2}^{2}L_{3}\right)U_{CA}^{2}\varepsilon_{0}}{48{\left(g_{0}-v_{g}\right)}^{2}g_{0}I}$$

Since the test structure is wide and the Poisson’s ratio *ṽ* is not considered in the theoretical model, Young’s modulus given by Equation (12) is actually the effective Young modulus *Ẽ* [23,24,25]. The relationship between the effective Young modulus and Young’s modulus is:

(13) $$\tilde{E}=\frac{E}{1-{\tilde{\nu }}^{2}}$$

The effective Young modulus can be obtained by substituting Equation (6) into Equation (12):

(14) $${\tilde{E}}_{1}=\frac{L_{2}\left(8L_{1}^{3}+18L_{1}^{2}L_{2}+12L_{1}L_{2}^{2}+3L_{2}^{3}+12L_{1}^{2}L_{3}+12L_{1}L_{2}L_{3}+4L_{2}^{2}L_{3}\right)U_{CA}^{2}\varepsilon_{0}}{4{\left(g_{0}-v_{g}\right)}^{2}g_{0}h_{1}^{3}}$$

where *Ẽ*_1_ is the effective Young modulus of the polysilicon layer and *U_CA_* is the contact voltage of test structure A.

### 2.3. Theoretical Model of Test Structure B with a Bilayer Beam

**Figure 3:**
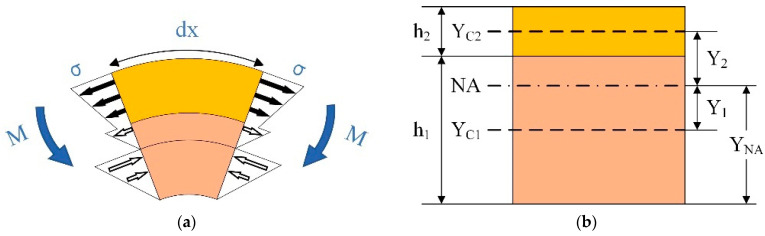
Schematic diagram of moment of inertia: (**a**) stress discontinuity in the bilayer film; (**b**) the neutral axis of the bilayer film.

The deflection of a cantilever beam with a bilayer film is similar to that with a single-layer film. The main difference is the moment of inertia. The calculation of the moment of inertia of the bilayer film is more complicated than that of the single-layer film, owing to a different Young’s modulus and the thickness of different layers, as illustrated in Figure 3a.

#### 2.3.1. Derivation of Neutral Axis

In case of the bilayer films, the strain is assumed to be continuous, whereas the stress is discontinuous as a result of different Young’s moduli of different layers. The so-called neutral axis (NA) refers to the location where the bending stress is zero. It is necessary to determine the NA for the deflection calculation of the bilayer film. The location of the NA is determined by Young’s modulus and the thickness of two layers. For the single-layer film, the NA is the centroid of the cross section. However, this is not the case for the bilayer film. As shown in Figure 3b, the distance from the NA to the bottom surface of the bilayer film is defined as *Y_NA_*; the centroids of the lower layer and the upper layer are defined as *Y_C_*_1_ and *Y_C_*_2_, respectively; and *Y*_1_ and *Y*_2_ are the distances from the NA to *Y_C_*_1_ and *Y_C_*_2_, respectively.

In a bent bilayer beam, the cross-section should be balanced and the resultant force of the cross section in the *x*-axis direction should be zero. This relationship can be described as:

(15) $$\sum F_{x}=0=\int \sigma dA=\int_{A_{1}}\sigma_{1}dA+\int_{A_{2}}\sigma_{2}dA$$

where *F_x_* is the axial force; *σ* represents the stress in the *x*-axis direction; A stands for the cross-section area; *σ*_1_ and *σ*_2_ are the axial stresses of the lower layer and the upper layer, respectively; and *A*_1_ and *A*_2_ are the cross-section areas of the lower layer and the upper layer, respectively.

The relationship between the axial stress and the bending curvature is shown in Equation (16):

(16) $$\sigma =-\frac{Ey}{\rho }$$

where *y* is the distance to the NA and *ρ* denotes the radius of curvature. Equation (17) is obtained by substituting Equation (16) into Equation (15):

(17) $$0=\int_{A_{1}}-\frac{E_{1}y_{1}}{\rho }dA+\int_{A_{2}}-\frac{E_{2}y_{2}}{\rho }dA$$

where *y*_1_ and *y*_2_ are the distances to NA in the lower layer and the upper layer, respectively; *E*_1_ is the Young modulus of the lower layer; and *E*_2_ is the Young modulus of the upper layer. 

In order to simplify the derivation, the bilayer film is assumed as ideally curved. Since *ρ* is constant, Equation (17) can be rewritten as:

(18) $$0=E_{1}\int_{A_{1}}y_{1}dA+E_{2}\int_{A_{2}}y_{2}dA$$

This can be simplified to:

(19) $$\begin{array}{ll} 0 & =E_{1}\left(Y_{1}A_{1}\right)+E_{2}\left(Y_{2}A_{2}\right)\\ & =E_{1}Y_{1}h_{1}+E_{2}Y_{2}h_{2} \end{array}$$

Both *Y*_1_ and *Y*_2_ are determined by *Y_NA_*, shown as:

(20) $$\begin{array}{ll} Y_{1} & =\frac{h_{1}}{2}-Y_{NA}\\ Y_{2} & =h_{1}+\frac{h_{2}}{2}-Y_{NA} \end{array}$$

*Y_NA_* is obtained by simultaneously solving Equations (19) and (20):

(21) $$Y_{NA}=\frac{E_{1}h_{1}^{2}+2E_{2}h_{1}h_{2}+E_{2}h_{2}^{2}}{2\left(E_{1}h_{1}+E_{2}h_{2}\right)}$$

#### 2.3.2. Displacement Calculation of the Bilayer Beam

Similar to the bending moment equation for the single-layer beam, the bending moment equation for the bilayer beam is given by:

(22) $$M=\frac{E_{1}}{\rho }\int_{A_{1}}y_{1}^{2}dA+\frac{E_{2}}{\rho }\int_{A_{2}}y_{2}^{2}dA$$

Notice that the integral is the second moment of the area, and Equation (22) can be simplified as:

(23) $$M=\frac{E_{1}I_{1}}{\rho }+\frac{E_{2}I_{2}}{\rho }$$

with

(24) $$\begin{array}{c} I_{1}=\int_{A_{1}}{\left(y-y_{NA}\right)}^{2}dA=W_{b}\frac{h_{1}\left[E_{1}^{2}h_{1}^{4}+2E_{1}E_{2}h_{1}^{3}h_{2}+E_{2}^{2}h_{2}^{2}\left(4h_{1}^{2}+6h_{1}h_{2}+3h_{2}^{2}\right)\right]}{12{\left(E_{1}h_{1}+E_{2}h_{2}\right)}^{2}}\\ I_{2}=\int_{A_{2}}{\left(y-y_{NA}\right)}^{2}dA=W_{m}\frac{h_{2}\left[2E_{1}E_{2}h_{1}h_{2}^{3}+E_{2}^{2}h_{2}^{4}+E_{1}^{2}h_{1}^{2}\left(3h_{1}^{2}+6h_{1}h_{2}+4h_{2}^{2}\right)\right]}{12{\left(E_{1}h_{1}+E_{2}h_{2}\right)}^{2}} \end{array}$$

where *I*_1_ and *I*_2_ are the moments of inertia of the lower layer and the upper layer, respectively.

In the left part of test structure B where *L*_1_ and *L*_2_ are located, Equation (1) is rewritten as:

(25) $$\frac{d^{2}v}{dx^{2}}=\frac{1}{\rho }=\frac{M}{E_{1}I_{1}+E_{2}I_{2}}$$

By replacing *EI* with *E*_1_*I*_1_ + *E*_2_*I*_2_ into Equation (4), the vertical displacement at the free end of the bilayer beam can be calculated as follows:

(26) $$\begin{array}{ll} \delta_{b} & =\delta_{2}+\theta_{b}L_{3}\\ & =\frac{q\left[3{\left(L_{1}+L_{2}\right)}^{4}-4L_{1}^{3}\left(L_{1}+L_{2}\right)+L_{1}^{4}\right]}{24\left(E_{1}I_{1}+E_{2}I_{2}\right)}+\frac{qL_{3}\left[{\left(L_{1}+L_{2}\right)}^{3}-L_{1}^{3}\right]}{6\left(E_{1}I_{1}+E_{2}I_{2}\right)}\\ & =\frac{qL_{2}\left[8L_{1}^{3}+12L_{1}L_{2}\left(L_{2}+L_{3}\right)+6L_{1}^{2}\left(3L_{2}+2L_{3}\right)+L_{2}^{2}\left(3L_{2}+4L_{3}\right)\right]}{24\left(E_{1}I_{1}+E_{2}I_{2}\right)} \end{array}$$

#### 2.3.3. Derivation of the Relationship between the Contact Voltage and the Effective Young Modulus

Similar to test structure A, the vertical displacement of the *L*_2_ region of test structure B is considered as a constant value *v_g_*, which is estimated by Equation (10). For a bilayer cantilever beam, Equation (8) is modified as:

(27) $$q=\frac{24\left(E_{1}I_{1}+E_{2}I_{2}\right)g_{0}}{L_{2}\left[8L_{1}^{3}+12L_{1}L_{2}\left(L_{2}+L_{3}\right)+6L_{1}^{2}\left(3L_{2}+2L_{3}\right)+L_{2}^{2}\left(3L_{2}+4L_{3}\right)\right]}$$

By substituting Equation (27) into Equation (7), the following equation can be found:

(28) $$\frac{24\left(E_{1}I_{1}+E_{2}I_{2}\right)g_{0}}{L_{2}\left[8L_{1}^{3}+12L_{1}L_{2}\left(L_{2}+L_{3}\right)+6L_{1}^{2}\left(3L_{2}+2L_{3}\right)+L_{2}^{2}\left(3L_{2}+4L_{3}\right)\right]}=W_{b}\frac{\varepsilon_{0}\cdot U^{2}}{2\cdot g^{2}}$$

Equation (29) is obtained by substituting Equation (24) and Equation (11) into Equation (28):

(29) $$\begin{array}{cc} \frac{W_{b}U_{CB}^{2}\varepsilon_{0}}{2{\left(g_{0}-v_{g}\right)}^{2}}= & \frac{2g_{0}\left({\tilde{E}}_{1}^{3}h_{1}^{5}+2{\tilde{E}}_{1}^{2}{\tilde{E}}_{2}h_{1}^{4}h_{2}\right)+4{\tilde{E}}_{1}{\tilde{E}}_{2}^{2}h_{1}^{3}h_{2}^{2}+6{\tilde{E}}_{1}{\tilde{E}}_{2}^{2}h_{1}^{2}h_{2}^{3}+3{\tilde{E}}_{1}{\tilde{E}}_{2}^{2}h_{1}h_{2}^{4}W_{b}}{{\left({\tilde{E}}_{1}h_{1}+{\tilde{E}}_{2}h_{2}\right)}^{2}L_{2}\left[8L_{1}^{3}+12L_{1}L_{2}\left(L_{2}+L_{3}\right)+6L_{1}^{2}\left(3L_{2}+2L_{3}\right)+L_{2}^{2}\left(3L_{2}+4L_{3}\right)\right]}+\\ & \frac{2g_{0}\left(3{\tilde{E}}_{1}^{2}{\tilde{E}}_{2}h_{1}^{4}h_{2}+6{\tilde{E}}_{1}^{2}{\tilde{E}}_{2}h_{1}^{3}h_{2}^{2}+4{\tilde{E}}_{1}^{2}{\tilde{E}}_{2}h_{1}^{2}h_{2}^{3}+2{\tilde{E}}_{1}{\tilde{E}}_{2}^{2}h_{1}h_{2}^{4}+{\tilde{E}}_{2}^{3}h_{2}^{5}\right)W_{m}}{{\left({\tilde{E}}_{1}h_{1}+{\tilde{E}}_{2}h_{2}\right)}^{2}L_{2}\left[8L_{1}^{3}+12L_{1}L_{2}\left(L_{2}+L_{3}\right)+6L_{1}^{2}\left(3L_{2}+2L_{3}\right)+L_{2}^{2}\left(3L_{2}+4L_{3}\right)\right]} \end{array}$$

where *U_CB_* is the contact voltage of test structure B and *Ẽ*_1_ is the effective Young modulus of the polysilicon layer obtained from test structure A. The effective Young modulus of the metal layer *Ẽ*_2_ can be determined by numerical calculation using Equation (29).

## 3. Finite Element Analysis

### 3.1. Simulation Method

In order to verify the theoretical model, the finite element analysis software COMSOL Multiphysics 5.6 (COMSOL Co., Ltd., Stockholm, Sweden) is used for simulation. The input parameters of dimensions in the simulation are listed in Table 2.

The finite element simulation is carried out with the electromechanical coupling multi-physics field, in which the electrostatic field contains the polysilicon layer of the cantilever beam, the drive electrode, and the air. Since the electric field mainly exists between the polysilicon layer of the cantilever beam and the drive electrode, the electrostatic field does not include the metal layer of the cantilever beam. The polysilicon layer of the cantilever beam is set as voltage terminal 1 and the voltage is set to be 0 V; the drive electrode is set as voltage terminal 2 and the voltage is set as the driving voltage U; and the air is set as independent non-solid charge conservation. The solid mechanics physical field contains two parts, i.e., the cantilever beam and the drive electrode. The bottom surface of the drive electrode and the cross section of the cantilever beam connected to the anchor are set as fixed constraints. The air is set as a deformable moving mesh. Both test structure A and test structure B are simulated using 3D models.

In order to avoid the pull-in phenomenon by using the steady-state simulation, the transient simulation method is adopted. In order to avoid the effects of kinetic energy when the step voltage is applied, the driving voltage is linearly applied in the transient simulation, as demonstrated in the experiment. Considering that the response relaxation time of the designed test structure listed in Table 2 is less than 0.1 s, and no air damping is applied in the simulation, the total rise time of the driving voltage is set to be 1 s. After the rise, a 0.1 s voltage hold time is set for structure stabilization. The results at 1.1 s are recorded.

### 3.2. Simulation Results

**Figure 4:**
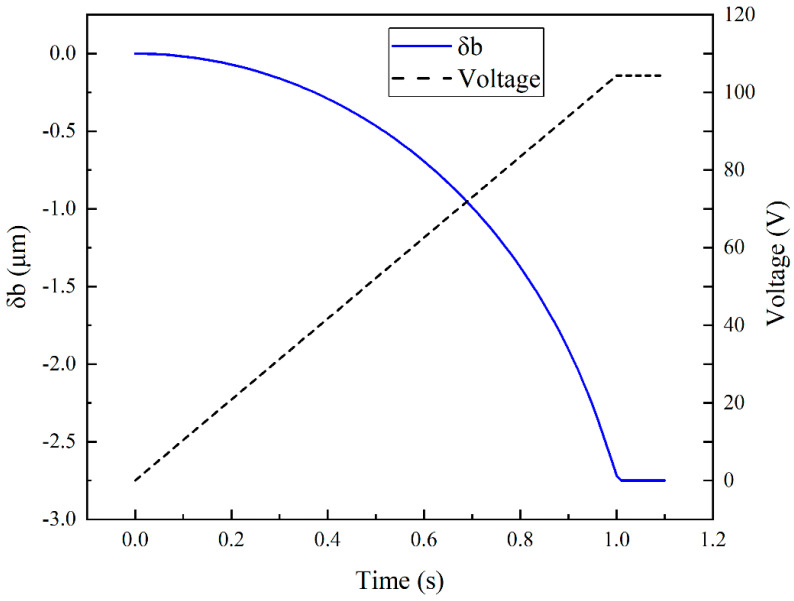
Transient vertical displacement at the free end of the cantilever beam and the driving voltage.

The simulation result of transient response of test structure A is illustrated in Figure 4. The vertical displacement curve at the free end of the cantilever beam shown in the figure indicates that the voltage and time settings of the transient simulation meet the simulation requirements.

**Figure 5:**
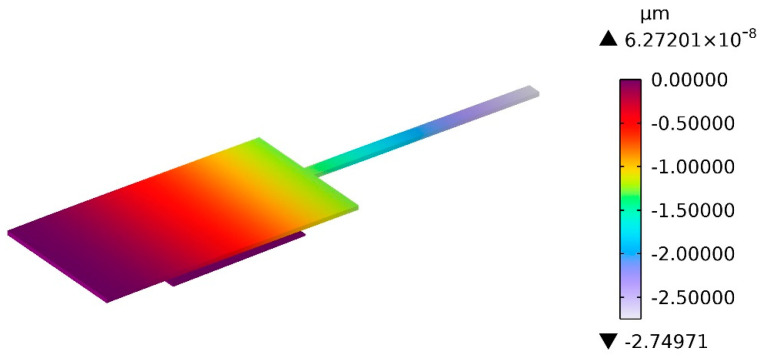
Simulation results of test structure A.

Eight types of test structure A with different dimensions listed in Table 2 are simulated. The simulation result of vertical displacement of test structure 1-A at 1.1 s is demonstrated in Figure 5. The contact voltage *U_CA_* and the maximum free-end displacement *δ_bA_* obtained by simulation are presented in Table 3. Considering that the influence of Poisson’s ratio on the test structure in simulation may be different from the classical theory of mechanics of materials, a simulated effective Young modulus with the zero Poisson ratio is introduced for verification purposes and the input effective Young modulus is calculated from the classical theory by using input simulation parameters. In the simulation, the input Young modulus E_1-In_ and the Poisson ratio *ṽ*_1_ of polysilicon are set to be 160 GPa and 0.22, respectively, and the vacuum dielectric constant ε_0_ is set as 8.85 × 10^−12^ F/m; accordingly, the input effective Young modulus of polysilicon (*Ẽ*_1-*EQ*_) calculated from Equation (13) is equal to 168.138 GPa. The values of the effective Young modulus of polysilicon (*Ẽ*_1_) estimated from the proposed theory model by substituting simulation results of U_CA_ into Equation (14) are shown in Table 3. The error between *Ẽ*_1_ and *Ẽ*_1-*EQ*_ is less than ±7%. The simulation is rerun with Poisson’s ratio set as zero. The simulated effective Young modulus *Ẽ*_1-*FEA*_ is then obtained as 164.430 Gpa. The error between *Ẽ*_1_ and *Ẽ*_1-*FEA*_ is less than ±5%. 

**Figure 6:**
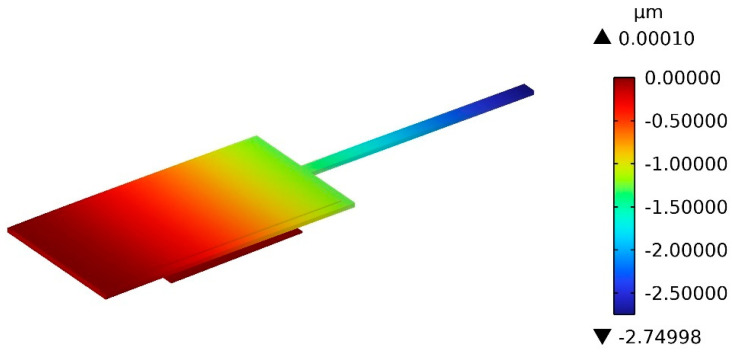
Simulation results of test structure B.

The simulation of test structure B is performed by the same procedure as the simulation of test structure A. All eight types of test structure B with different dimensions given in Table 2 are simulated. The result of the vertical displacement of test structure 1-B at 1.1 s is displayed in Figure 6. The simulation results of the contact voltage *U_CB_* and the free-end displacement *δ_bB_* are shown in Table 4. The input Young modulus *E*_2-*In*_ and Poisson’s ratio *ṽ*_2_ of Au are set to be 70 GPa and 0.44, respectively, and the input effective Young modulus of Au (*Ẽ*_2-*EQ*_) calculated by using Equation (13) is 86.806 GPa. The results of the effective Young modulus of Au (Ẽ_2_) extracted from the proposed theory model by using Equation (29) are listed in Table 4. The error between *Ẽ*_2_ and *Ẽ*_2-*In*_ is less than ±11%. The simulated effective Young modulus *Ẽ*_2-*FEA*_ is 79.600 Gpa when the Poisson’s ratio is set to be zero. The error between *Ẽ*_2_ and *Ẽ*_2-*FEA*_ is less than ±3%.

As shown in Table 3 and Table 4, eight groups of results determined from the theoretical model are compared with the input effective Young modulus calculated by Equation (13), and the error is less than ±11%; the results are also compared with the simulated effective Young modulus, and the error is less than ±5%. As a result, the finite element simulation results verify the established theoretical model.

## 4. Experiments and Discussion

**Figure 7:**
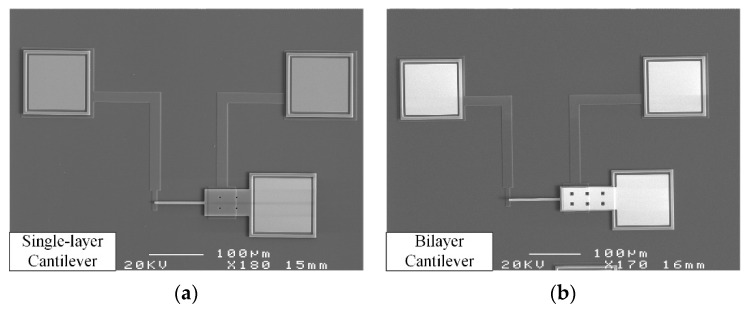
SEM photograph of the test structure: (**a**) test structure A; (**b**) test structure B.

The samples of test structure A and test structure B of different sizes listed in Table 2 are fabricated by using the standard surface-micromachining process from PolyMUMPs (MEMSCAP Inc., Durham, NC, USA). The structural layers in the process include poly0, poly1, poly2, and metal layers, and the sacrificial layers include the first oxide between poly0 and poly1 and the second oxide between poly1 and poly2. The poly0, poly1, and poly2 layers are made of polysilicon fabricated by using low-pressure chemical vapor deposition (LPCVD); the first oxide and second oxide layers are made of phosphosilicate glass (PSG) fabricated by using LPCVD; and the metal layer is made of Au fabricated by sputtering. The drive electrode and contact electrode are fabricated by poly0; the single-layer cantilever beam of test structure A is fabricated by poly2; the bilayer cantilever beam of test structure B is fabricated by poly2 and metal; and the sacrificial layer is fabricated by combined first oxide and second oxide layers. The SEM image of the test structures are shown in Figure 7.

**Figure 8:**
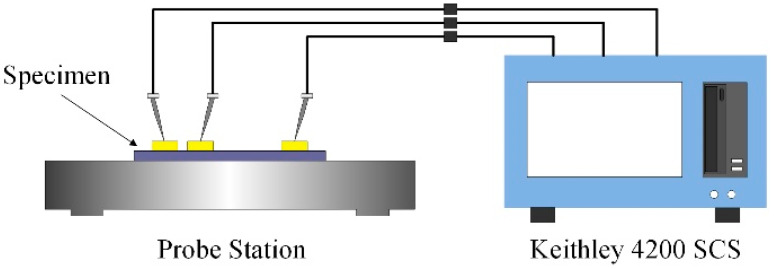
Schematic diagram of the experiment equipment.

The experiment is performed by using a DC probe station and a Keithley 4200-scs semiconductor characterization system, as shown in Figure 8. A voltage sweep from 0 V to V_d_ with an increment of 0.1 V is applied between the drive electrode and the cantilever beam, while the current between the cantilever beam and the contact electrode is monitored as the criterion for contact. When contact occurs, a sudden step in the current is detected and the applied voltage is recorded. The strategy of the experiment is upward search. V_d_ is set to 80% of U_CA_ in Table 3 and U_CB_ in Table 4, and the upward search strategy is used with an increment of 0.1 V until V_d_ reaches 200 V, which is the upper limit of the Keithley 4200-scs semiconductor characterization system. If no contact is detected, it will be recorded as no result. 

**Figure 9:**
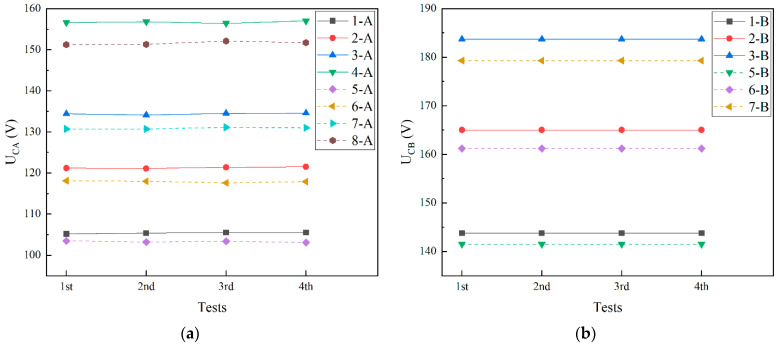
Measure results of test structures: (**a**) test structure A; (**b**) test structure B.

Figure 9 displays the measured contact voltages of test structure A (*U_CA_*) and test structure B (*U_CB_*) with different dimensions. Each structure is tested for four times. It can be seen that the repeatability of the test structure is good. To investigate the influence of different dimensions on the test results, the test structures are divided into four groups. With regard to the influence of beam length on the test results, test structures 1 and 5, 2 and 6, 3 and 7, and 4 and 8 are set as one group, respectively. The beam lengths of each group are different while the other dimensions are the same. It can be seen from Figure 9 that the influence of the beam length on the contact voltage is relatively small, and the longer the length, the smaller the contact voltage. As for the influence of the drive electrode length on the test results, test structures 1 and 2, 3 and 4, 5 and 6, and 7 and 8 are set as one group, respectively. The drive electrode lengths of each group are different while the other dimensions are the same. Figure 9 shows that the effect of the drive electrode length on the contact voltage is relatively large. The longer the drive electrode, the smaller the contact voltage. When examining the influence of the drive electrode location on the test results, test structures 1 and 3, 2 and 4, 5 and 7, and 6 and 8 are set as one group, respectively. The drive electrode locations of each group are different while the other dimensions are the same. As shown in Figure 9, the bigger the distance between the drive electrode and the anchor, the smaller the contact voltage. The drive electrode location has the greatest influence on the contact voltage. The average values of *U_CA_* and *U_CB_* in the experiment are summarized in Table 5. The effective Young modulus of polysilicon (Ẽ_1_) is determined from Equation (14) by using the average measured values of *U_CA_*; the effective Young modulus of Au (*Ẽ*_2_) is estimated from Equation (29) by using the average measured values of *U_CB_*. Young’s moduli *E*_1_ and *E*_2_ in Table 5 are extracted from the effective Young modulus using Equation (13).

The average measured value of Young’s moduli for the surface-micro-machined polysilicon film is 153.65 GPa with a standard deviation of 0.81 GPa. The errors compared with the existing values reported in [28,30,32] are less than 10%, and the results are in agreement with [26,33]. The average effective Young modulus of the surface-micro-machined Au film is measured to be (73.50 ± 1.16) GPa. As compared to [27,29,31], the errors are less than 10%. In comparison with [13,14], the errors of Young’s moduli for polysilicon and Au are both less than 5%, since the fabrication process is the same. This verifies the proposed extraction method.

There are several factors that may influence the experimental results and limit the applications of the test structure. First, the intensity *q* is assumed as a constant in order to simplify the extraction equation. This may cause an error because the vertical displacement of the *L*_2_ region is not identical and *q* should be a function of *x*. To minimize the error, we adopt a derived mean value to estimate the vertical displacement and the accuracy of the model is improved by 5%. Second, the stiffness of the polysilicon layer is much larger than the Au layer, since Young’s modulus and the thickness of the polysilicon layer are both larger than the Au layer. Consequently, *E*_1_ obtained from test structure A will significantly affect *E*_2_ obtained from test structure B. In other words, *E*_2_ is more sensitive to error. Therefore, the test structure is more reliable for composite thin films with similar stiffness. Third, it is believed that the residual stress of single-layer cantilever is released. However, this is not the case for the bilayer film. The warpage is observed in bilayer cantilevers longer than 200 μm due to the existence of the stress gradient between two layers. As a result, the initial gap between the cantilever beam and the bottom drive electrode is uncertain, which would significantly influence the contact voltage *U_CB_*. This indicates that the proposed method is more suitable for composite thin films with small stress gradients. In our experiments, specimens with flat beams are used. Thus, the experimental results listed in Table 5 are valid. 

As demonstrated above, test structures for an Au/polysilicon bilayer beam are fabricated and tested as a typical example. The proposed method can be extended to three or more layers and applied to insulating films as long as one layer of the multi-layer thin films is a conducting film. The derived formulae are applicable when the conducting layer is the lowest layer of the multilayer thin films. However, if there is one or more dielectric layers between the conducting layer and the bottom electrode, the derived formulae need to be modified. This will animate our future work.

## 5. Conclusions

A test structure for measuring Young’s modulus of composite thin films is designed. The theoretical model of the test structure is established, and the model is verified by simulation. The simulation results indicate that the error of the established theoretical model is less than 5% and 12% in comparison with the simulated effective Young modulus and the input effective Young modulus is calculated by Equation (13), respectively. The designed test structures are fabricated by PolyMumps and the experiment is performed. The error between the effective Young modulus measured by the experiment and the related literature is less than 10%. The proposed method is simple and relatively precise with good repeatability and low requirements for equipment, making is suitable for PCM in the MEMS fabrication process.

## Figures and Tables

**Table 1:** List of constants for Equation (10).

a_1_	a_2_	a_3_	a_4_	a_5_	a_6_	a_7_	a_8_	a_9_	a_10_
512	2560	6240	$\frac{48192}{5}$	$\frac{51244}{5}$	$\frac{38616}{5}$	$\frac{87050}{21}$	$\frac{32780}{21}$	$\frac{21718}{55}$	$\frac{43436}{715}$

**Table 2:** Dimensions of test structures A and B.

No.	*L_b_* (μm)	*L_eoff_* (μm)	*L_e_* (μm)	*W_b_* (μm)	*W_m_* (μm)	*W_eoff_* (μm)	*W_moff_* (μm)	*g*_0_ (μm)	*h*_0_ (μm)	*h*_1_ (μm)	*h*_2_ (μm)
1	180	50	50	50	44	5	3	2.75	0.5	1.5	0.5
2	180	50	40	50	44	5	3	2.75	0.5	1.5	0.5
3	180	40	50	50	44	5	3	2.75	0.5	1.5	0.5
4	180	40	40	50	44	5	3	2.75	0.5	1.5	0.5
5	200	50	50	50	44	5	3	2.75	0.5	1.5	0.5
6	200	50	40	50	44	5	3	2.75	0.5	1.5	0.5
7	200	40	50	50	44	5	3	2.75	0.5	1.5	0.5
8	200	40	40	50	44	5	3	2.75	0.5	1.5	0.5

**Table 3:** Finite element simulation results of test structure A.

No.	*E*_1-*In*_ (GPa)	*ṽ*_1_	*Ẽ*_1-*EQ*_ (GPa)	*Ẽ*_1-*FEA*_ (GPa)	*U_CA_* (V)	*δ_bA_* (μm)	*Ẽ*_1_ (GPa)	*Error_1_*_-*EQ*_	*Error_1_*_-*FEA*_
1-A	160	0.22	168.138	164.430	104.33	2.74971	157.889	−6.10%	−3.98%
2-A	160	0.22	168.138	164.430	119.99	2.75015	157.594	−6.27%	−4.16%
3-A	160	0.22	168.138	164.430	133.29	2.74998	158.090	−5.98%	−3.86%
4-A	160	0.22	168.138	164.430	154.09	2.74980	157.251	−6.47%	−4.37%
5-A	160	0.22	168.138	164.430	102.09	2.75019	158.320	−5.84%	−3.72%
6-A	160	0.22	168.138	164.430	116.97	2.74975	157.577	−6.28%	−4.17%
7-A	160	0.22	168.138	164.430	129.24	2.75019	157.700	−6.21%	−4.09%
8-A	160	0.22	168.138	164.430	149.26	2.75028	157.241	−6.48%	−4.37%

**Table 4:** Finite element simulation results of test structure B.

No.	*E*_2-*In*_ (GPa)	*ṽ*_2_	*Ẽ*_2-*EQ*_ (GPa)	*Ẽ*_2-*FEA*_ (GPa)	*U_CB_* (V)	*δ_bB_* (μm)	*Ẽ*_2_ (GPa)	*Error*_2-*EQ*_	*Error*_2-*FEA*_
1-B	70	0.44	86.806	79.600	135.60	2.74998	77.4613	−10.76%	−2.69%
2-B	70	0.44	86.806	79.600	156.02	2.75004	77.8497	−10.32%	−2.20%
3-B	70	0.44	86.806	79.600	173.34	2.74998	77.8627	−10.30%	−2.18%
4-B	70	0.44	86.806	79.600	200.60	2.75000	77.9781	−10.17%	−2.04%
5-B	70	0.44	86.806	79.600	132.59	2.75003	77.7396	−10.44%	−2.34%
6-B	70	0.44	86.806	79.600	151.94	2.74999	77.3546	−10.89%	−2.82%
7-B	70	0.44	86.806	79.600	168.17	2.74999	78.0734	−10.06%	−1.92%
8-B	70	0.44	86.806	79.600	194.29	2.74970	77.9237	−10.23%	−2.11%

**Table 5:** Experimental results of test structures.

No.	*U_CA_* (V)	*U_CB_* (V)	*Ẽ*_1_ (GPa)	*Ẽ*_2_ (GPa)	*E*_1_ (GPa)	*E*_2_ (GPa)
1	105.4	143.8	161.145	90.451	153.346	72.940
2	121.3	165	161.054	89.031	153.259	71.794
3	134.4	183.7	160.734	91.063	152.954	73.433
4	156.7	N.A. *	162.623	N.A.	154.752	N.A.
5	103.3	141.5	162.095	92.858	154.250	74.880
6	117.9	161.2	160.093	90.851	152.344	73.262
7	130.9	179.3	161.777	92.659	153.947	74.720
8	151.6	N.A.	162.210	N.A.	154.359	N.A.
References					149.3–171.5 [26]	79 [27]
					160 [28]	74 [29]
					163 [30]	77.2 [31]
					156.3 ± 2.6 [32]	75.72 [13]
					151.78 [13]	70.72–75.34 [14]
					151.38–154.93 [14]	

* N.A. represents for not available, which means no contact is found within the voltage upper limit of the experiment equipment.
